# Disparities in the Surgical Management of the Axilla by Self-Identified Race in the Multicenter Neoadjuvant I-SPY2 Trial

**DOI:** 10.1245/s10434-025-17864-y

**Published:** 2025-07-23

**Authors:** Mandeep Kaur, Katrina Dimitroff, Judy C. Boughey, Laura J. Esserman, Christina Yau, Julia Tchou, Astrid Quirarte, Marie Catherine Lee, Marissa M. Howard-McNatt, Kayla Switalla, Henry Kuerer, Candice Sauder, Lauren M. Postlewait, Cletus Arciero, Roshni Rao, Anne Wallace, Chantal Reyna, Kamran Ahmed, Lily Gutnik, Neil Taunk, Jane Perlmutter, Angela DeMichele, Douglas Yee, Nola M. Hylton, W. Fraser Symmans, Hope S. Rugo, Rebecca A. Shatsky, Claudine Isaacs, Sonali Rudra, Paula Pohlmann, Cheryl Ewing, Jasmine Wong, Michael Alvarado, Nora Jaskowiak, Nicolas Prionas, Mehra Golshan, Mara A. Piltin, Olufunmilayo I. Olopade, Rita A. Mukhtar

**Affiliations:** 1 School of Medicine, University of California San Francisco, San Francisco, CA USA; 2 University of California San Francisco, San Francisco, CA USA; 3 Department of Surgery, Mayo Clinic, Rochester, MN USA; 4 Department of Surgery, University of California San Francisco, San Francisco, CA USA; 5 University of Pennsylvania, Philadelphia, Pennsylvania USA; 6 Comprehensive Breast Program, Moffitt Cancer Center, Tampa, FL USA; 7 Department of Surgical Oncology, Wake Forest, Winston-Salem, NC USA; 8 Department of Breast Surgical Oncology, MD Anderson Cancer Center, Houston, TX USA; 9 Department of Surgery, UC Davis Health Comprehensive Cancer Center, Sacramento, CA USA; 10 Division of Surgical Oncology, Department of Surgery, Winship Cancer Institute, Emory University, Atlanta, GA USA; 11 Department of Surgery, Columbia University Medical Center, New York, NY USA; 12 Department of Surgery, University of California San Diego, San Diego, CA USA; 13 Department of Surgery, Loyola University Medical Center, Chicago, IL USA; 14 Department of Radiation Oncology, H. Lee Moffitt Cancer Center and Research Institute, Tampa, FL USA; 15 Department of Surgery, University of Alabama at Birmingham, Birmingham, AL USA; 16 Department of Radiation Oncology, University of Pennsylvania, Philadelphia, PA USA; 17 Gemini Group, Ann Arbor, MI USA; 18 Perelman School of Medicine, University of Pennsylvania, Philadelphia, PA USA; 19 Masonic Cancer Center, University of Minnesota, Minneapolis, MN USA; 20 Department of Radiology, University of California, San Francisco, San Francisco, CA USA; 21 Department of Pathology, University of Texas MD Anderson Cancer Center, Houston, TX USA; 22 Department of Medicine, University of California San Francisco, San Francisco, CA USA; 23 Moores Cancer Center, University of California San Diego, La Jolla, San Diego, CA USA; 24 Lombardi Comprehensive Cancer Center, Georgetown University, Washington, DC USA; 25 Department of Surgery, MedStar Georgetown University, Washington, DC USA; 26 Department of Surgery, University of Chicago, Chicago, IL USA; 27 Department of Radiation Oncology, University of California San Francisco, San Francisco, CA USA; 28 Department of Surgery, Yale School of Medicine, New Haven, CT USA; 29 Division of Breast and Melanoma Surgical Oncology, Department of Surgery, Mayo Clinic, Rochester, MN USA; 30 Center for Clinical Cancer Genetics and Global Health, University of Chicago, Chicago, IL USA

## Abstract

**Background:**

Neoadjuvant chemotherapy (NAC) may allow de-escalation of axillary surgery; yet treatment disparities persist. We aimed to assess race-based disparities in use of axillary lymph node surgery (ALND) among patients who achieve a nodal response in the context of a large, multicenter NAC trial.

**Methods:**

We conducted a retrospective analysis of the I-SPY 2 trial. All patients received NAC, but type of surgery was not mandated. Multivariable logistic regression was used to predict odds ratio (OR) of undergoing ALND by race while adjusting for clinical and demographic confounders, including age, region, tumor receptor subtype, clinical and pathologic node status (cN and ypN +/−, respectively), and clinical and pathologic tumor size (cT and ypT, respectively).

**Results:**

Among 1394 patients, 79.4% identified as White, 11.2% Black, and 9.4% Asian/other. More than half (52.5%) were cN+ at baseline, and 66.9% were ypN- after NAC, with no significant differences in nodal downstaging by race. Overall ALND rates were higher in Black patients (50.6%) compared to White (37.5%) and Asian/other (38.9%) patients (*p *= 0.007). Notably, among those who converted from cN+ to ypN−, Black patients underwent ALND more frequently (62%) than White (41.2%) and Asian/other (40%) patients (*p *= 0.021). In multivariable analysis, Black patients had 70% higher odds of undergoing ALND compared with White patients (OR 1.7, 95% confidence interval (CI) 1.09–2.66, *p *= 0.02).

**Conclusions:**

Despite no differences in nodal downstaging, Black patients in I-SPY 2 were significantly more likely to undergo ALND. These disparities may stem from unmeasured patient, provider, or systemic factors affecting surgical planning.

**Supplementary Information:**

The online version contains supplementary material available at 10.1245/s10434-025-17864-y.

Breast cancer treatment strategies have evolved significantly, with the use of neoadjuvant chemotherapy (NAC) allowing for the downstaging of nodal disease and a reduction in the need for axillary lymph node dissection (ALND).^1–5^ Axillary lymph node dissection is associated with significant morbidity, including lymphedema, pain, and decreased shoulder mobility.^6–10^ Despite the shift towards less invasive axillary management, disparities in ALND rates by race persist, with Black-identifying patients more likely to undergo ALND.^11–13^ Whether such disparities persist in the context of NAC and clinical trials is less well understood. We therefore sought to determine whether rates of ALND differ by race in a large, multi-center NAC clinical trial.

Given that clinical trials are designed to provide standardized treatment protocols and minimize variations in care, they present a unique opportunity to evaluate whether racial disparities persist even in a controlled setting. A prior investigation of the same NAC trial that is the focus of the current study found no race-based differences in pathologic complete response achievement.^14^ However, in an analysis of the National Cancer Database, among those who achieved downstaging after NAC, Black women were still found to receive higher ALND.^15^ While the data on race-based disparities in ALND after NAC are limited, this finding raises concern for factors beyond clinical indicators, such as patient preference, provider decision-making, or systemic bias, that may influence surgical choices.

In this study, we aimed to investigate differences in the rates of ALND by race in the context of a multicenter NAC trial to better understand the factors that drive disparities in the surgical management of the axilla. Because the trial does not mandate the type of axillary surgery, it provides a unique opportunity to examine variation in the adjuvant setting, where surgical decision-making may be influenced by factors beyond standardized protocol. By identifying patterns in the use and de-escalation of ALND across race groups after NAC, we seek to highlight potential contributors to inequities in care and inform efforts to standardize surgical treatment practices.

## Methods

I-SPY 2 is a prospective, adaptive trial for patients with molecularly high-risk clinical stage II-III breast cancer who are randomized to novel neoadjuvant chemotherapy (NAC) agents. The trial’s primary endpoint was rate of pathologic complete response (pCR). We retrospectively analyzed data from I-SPY 2 patients who completed NAC and surgical treatment during 2010–2021 across 19 participating centers. Type of axillary surgery was not mandated by the trial and was categorized as sentinel lymph node surgery (SLN)-only or axillary dissection (ALND) (± SLN), based on review of operative reports.

Self-identified race was collected and categorized as White, Black, or Asian/other, and self-identified ethnicity was categorized as Hispanic/Latino or non-Hispanic/Latino. Patient age and menopausal status at diagnosis was obtained from case report forms. Region of treatment was grouped as Midwest, Northeast, South, and West, based on the Centers for Disease Control and Prevention’s geographic region guidelines for the United States. Clinicopathologic characteristics collected include clinical stage (defined by AJCC guidelines and grouped as I/II and III), tumor histology (ductal or lobular), and tumor grade. Clinical and pathological nodal status and T category were collected as cN, ypN, cT, and ypT, respectively. Nodal status was grouped as positive or negative with cN1, cN2, and cN3 categories considered cN+ and cN0 category considered cN-; cN+ status required pre-treatment needle biopsy demonstrating nodal disease. Tumor receptor subtypes were classified by hormone receptor (HR) and human epidermal growth factor receptor 2 (HER) status, and grouped as HR+HER2−, triple-negative (HR−/HER2−), or HER2+. Cases missing data on type of axillary surgery were excluded.

### Statistical Analysis

The objective of this study was to compare ALND rates by self-identified race/ethnicity, stratified by clinical and pathological nodal status. We used chi-squared tests to evaluate relationships between race/ethnicity and factors, such as cN and ypN status, tumor receptor subtype, and region of treatment. To adjust for confounders, we used a multivariable logistic regression model including race, age, region of treatment, tumor receptor subtype, cN (+/−), cT (grouped T1-T2 and T3-T4), ypN (+/−), and ypT (grouped as T0/Tis, T1-T2, and T3-T4) to identify odds ratios (ORs) of undergoing ALND with 95% confidence intervals (CIs). Statistical analyses were performed using R version 4.3.1. Two-tailed *p* values < 0.05 were considered statistically significant.

## Results

### Overall Study Population

We identified 1394 patients in our final cohort. The average age was 48.3 ± 11.2 years, and 36.3% were postmenopausal. The most common region of treatment was the West (48.3%) (Table 1). Self-identified race was White in 1107 (79.4%), Black in 156 (11.2%), and Asian/other in 131 (9.4%), and 11.3% of all patients identified as Hispanic/Latino. Notably, we found a significant association between self-identified race and region of treatment. We found that Asian/other patients were significantly more often treated in the West, whereas Black patients were significantly more often treated in the South (78.6% of Asian/other patients treated in the West versus 49.2% White and 16% Black; 46.2% of Black patients treated in the South vs. 22.6% White and 8.4% Asian/other, *p* < 0.001).

**Table 1:** Study cohort characteristics shown overall and by self-identified race

Variable	Total (*N *= 1394)	White (*n *= 1107)	Black (*n *= 156)	Asian/other (*n *= 131)
Age (mean ± SD)	48.3 ± 11.2	48.5 ± 11.1	48.6 ± 11.8	46.1 ± 10.9
Hispanic ethnicity	157/1394 (11.3%)	149/1107 (13.5%)	3/156 (1.9%)	5/131 (3.8%)
Postmenopausal status	418/1150 (36.3%)	338/916 (36.9%)	37/110 (33.6%)	43/124 (34.7%)
*Region*				
West	673/1394 (48.3%)	545/1107 (49.2%)	25/ 56 (16%)	103/131 (78.6%)
South	333/1394 (23.9%)	250/1107 (22.6%)	72/ 56 (46.2%)	11/131 (8.4%)
Midwest	310/1394 (22.2%)	263/1107 (23.8%)	32/156 (20.5%)	15/131 (11.5%)
Northeast	78/1394 (5.6%)	49/1107 (4.4%)	27/156 (17.3%)	2/131 (1.5%)
Pathologic Complete Response	502/1394 (36%)	409/1107 (36.9%)	51/156 (32.7%)	42/131 (32.1%)
*Clinical stage*				
I/II	1023/1394 (73.4%)	807/1107 (72.9%)	111/156 (71.2%)	105/131 (80.2%)
III	371/1394 (26.6%)	300/1107 (27.1%)	45/156 (28.8%)	26/131 (19.8%)
*Tumor histology*				
Ductal	1205/1329 (90.7%)	950/1054 (90.1%)	136/148 (91.9%)	119/127 (93.7%)
Lobular	124/1329 (9.3%)	104/1054 (9.9%)	12/148 (8.1%)	8/127 (6.3%)
*Tumor grade*				
1	14/966 (1.4%)	11/793 (1.4%)	1/66 (1.5%)	2/107 (1.9%)
2	282/966 (29.2%)	240/793 (30.3%)	13/66 (19.7%)	29/107 (27.1%)
3	670/966 (69.4%)	542/793 (68.3%)	52/66 (78.8%)	76/107 (71%)
*cT category*				
1	36/1394 (2.6%)	27/1107 (2.4%)	3/156 (1.9%)	6/131 (4.6%)
2	938/1394 (67.3%)	747/1107 (67.5%)	102/156 (65.4%)	89/131 (67.9%)
3	368/1394 (26.4%)	291/1107 (26.3%)	43/156 (27.6%)	34/131 (26%)
4	52/1394 (3.7%)	42/1107 (3.8%)	8/156 (5.1%)	2/131 (1.5%)
cN positive	732/1394 (52.5%)	580/1107 (52.4%)	91/156 (58.3%)	61/131 (46.6%)
*cN category*				
0	662/1394 (47.5%)	527/1107 (47.6%)	65/156 (41.7%)	70/131 (53.4%)
1	589/1394 (42.3%)	465/1107 (42%)	75/156 (48.1%)	49/131 (37.4%)
2	62/1394 (4.4%)	47/1107 (4.2%)	8/156 (5.1%)	7/131 (5.3%)
3	81/1394 (5.8%)	68/1107 (6.1%)	8/156 (5.1%)	5/131 (3.8%)
*ypT category*				
T0/Tis	546/1391 (39.3%)	447/1104 (40.5%)	54/156 (34.6%)	45/131 (34.4%)
T1	435/1391 (31.3%)	333/1104 (30.2%)	57/156 (36.5%)	45/131 (34.4%)
T2	266/1391 (19.1%)	206/1104 (18.7%)	28/156 (17.9%)	32/131 (24.4%)
T3	125/1391 (9%)	105/1104 (9.5%)	12/156 (7.7%)	8/131 (6.1%)
T4	19/1391 (1.4%)	13/1104 (1.2%)	5/156 (3.2%)	1/131 (0.8%)
ypN positive	462/1394 (33.1%)	364/1107 (32.9%)	51/156 (32.7%)	47/131 (35.9%)
*Receptor subtype*				
HR+/HER2−	595/1394 (42.7%)	490/1107 (44.3%)	60/156 (38.5%)	45/131 (34.4%)
HR−/HER2−	471/1394 (33.8%)	352/1107 (31.8%)	70/156 (44.9%)	49/131 (37.4%)
HER2+	328/1394 (23.5%)	265/1107 (23.9%)	26/156 (16.7%)	37/131 (28.2%)

Data are numbers with percentages in parentheses

*SD* standard deviation; *c* clinical; *yp* pathologic; *Tis* in situ; *HR* hormone receptor; *HER2* human epidermal growth factor receptor 2; +, receptor status positive; −, receptor status negative

Patients were most commonly clinically stage I/II (73.4%) with grade 3 tumors (69.4%). Most tumors were ductal (90.7%), and the distribution of tumor receptor subtype was 42.7% HR+/HER2−, 33.8% HR−/HER2−, and 23.5% HER2+. For surgical treatment of the primary tumor, most patients underwent mastectomy (55.9% compared with 44.1% lumpectomy; Table 2).

**Table 2:** Type of breast surgery, axillary surgery, and nodes removed shown overall and by self-identified race

Variable	Total (*N *= 1394)	White (*n *= 1107)	Black (*n *= 156)	Asian/other (*n *= 131)
*Type of breast surgery*				
Lumpectomy	615/1394 (44.1%)	476/1107 (43%)	69/156 (44.2%)	70/131 (53.4%)
Mastectomy	779/1394 (55.9%)	631/1107 (57%)	87/156 (55.8%)	61/131 (46.6%)
*Type of axillary surgery*				
SLN-only	849/1394 (60.9%)	692/1107 (62.5%)	77/156 (49.4%)	80/131 (61.1%)
ALND	545/1394 (39.1%)	415/1107 (37.5%)	79/156 (50.6%)	51/131 (38.9%)
Total nodes removed at surgery	8.5 ± 8.4	8.5 ± 8.5	9.2 ± 8.1	8.5 ± 8.4
Total positive nodes removed at surgery	1.3 ± 3.3	1.3 ± 3.3	1.3 ± 2.6	1.5 ± 3.7

Data are numbers with percentages in parentheses

*SLN* sentinel lymph node surgery; *ALND* axillary lymph node dissection with or without SLN

### Nodal Status and Self-Identified Race

Overall, 52.5% of the study population was cN+ and 33.1% was ypN+. There were no differences in cN or ypN category by race or by Hispanic/Latino ethnicity. Among cN+ patients, the distribution of cN1 compared with cN2/3 disease did not differ by race, with the proportion of cN2/3 cases being 10.4%, 10.3%, and 9.2% in White, Black, and Asian/other patients respectively (*p *= 0.4). Most cN− patients remained ypN−, with 15% being found to have ypN+ disease at surgery. Conversely, among cN+ patients, 49.6% remained ypN+, whereas 50.4% had nodal response and were ypN− at surgery. The relationship between cN status and ypN status did not differ by race (Table 3). Particularly, the rate of conversion from cN+ to ypN− was 26.6%, 32.1%, and 19.1% for White, Black, and Asian/other, respectively (*p *= 0.3).

**Table 3:** Changes in clinical to pathologic node status by self-identified race

Variable	White (*n *= 1107)	Black (*n *= 156)	Asian/other (*n *= 131)	*p*
cN+ to ypN−	294/1107 (26.6%)	50/156 (32.1%)	25/131 (19.1%)	0.3
cN+ to ypN+	286/1107 (25.8%)	41/156 (26.3%)	36/131 (27.5%)	
cN− to ypN−	449/1107 (40.6%)	55/156 (35.3%)	59/131 (45%)	
cN− to ypN+	78/1107 (7%)	10/156 (6.4%)	11/131 (8.4%)	

Data are numbers with percentages in parentheses unless otherwise indicated

*cN* clinical node status; *ypN* pathologic node status; +, positive; −, negative

*N *= 1394

### Axillary Lymph Node Dissection Rates by Race and Regions

In the entire study cohort, 39.1% of patients underwent ALND and the remaining 60.9% underwent SLN-only). The rate of ALND did not significantly differ by ethnic identity (37.6% ALND in those who identified as Hispanic/Latino vs. 39.3%, *p *= 0.7). However, when evaluating ALND rates by self-identified race, Black patients had significantly higher rates of ALND compared to those identifying as White or Asian/Other (50.6%, 37.5%, and 38.9%, respectively, *p *= 0.007).

When stratified by nodal status, Black patients remained more likely to undergo ALND, specifically among the cN+ and ypN− subgroups (Table 4). Among cN+ patients, ALND was performed in 59.1%, 74.7%, and 60.7% of White, Black, and Asian/other patients respectively (*p *= 0.018). Among ypN− patients, ALND was performed in 19.2%, 33.3%, and 20.2% of White, Black, and Asian/other patients respectively (*p *= 0.004). For cN− and ypN+ subgroups, there was no difference in the rate of ALND by race. Despite no difference in nodal response rates by race, among those that converted from cN+ to ypN−, Black patients still had significantly higher rates of ALND than Asian/other or White patients (62% vs. 40% and 41.2%, respectively, *p *= 0.021).

**Table 4:** Associations between axillary surgery and self-identified race overall and by nodal status

Variable	White (*n *= 1107)	Black (*n *= 156)	Asian/other (*n *= 131)	*p*
Overall^1^				0.007
SLN-only	692/1107 (62.5%)	77/156 (49.4%)	80/131 (61.1%)	
ALND	415/1107 (37.5%)	79/156 (50.6%)	51/131 (38.9%)	
cN−^2^				0.3
SLN-only	455/527 (86.3%)	54/65 (83.1%)	56/70 (80%)	
ALND	72/527 (13.7%)	11/65 (16.9%)	14/70 (20%)	
cN+^3^				0.018
SLN-only	237/580 (40.9%)	23/91 (25.3%)	24/61 (39.3%)	
ALND	343/580 (59.1%)	68/91 (74.7%)	37/61 (60.7%)	
ypN−^4^				0.004
SLN-only	600/743 (80.8%)	70/105 (66.7%)	67/84 (79.8%)	
ALND	143/743 (19.2%)	35/105 (33.3%)	17/84 (20.2%)	
ypN+^5^				0.2
SLN-only	92/364 (25.3%)	7/51 (13.7%)	13/47 (27.7%)	
ALND	272/364 (74.7%)	44/51 (86.3%)	34/47 (72.3%)	
cN+ to ypN-^6^				
SLN-only	173/294 (58.8%)	19/50 (38%)	15/25(60%)	0.021
ALND	121/294 (41.2%)	31/50 (62%)	10/25 (40%)	

Data are numbers with percentages in parentheses unless otherwise indicated

*SLN* sentinel lymph node surgery; *ALND* axillary lymph node dissection with or without SLN; *cN* clinical node status; *ypN* pathologic node status; +, positive; −, negative

Type of axillary surgery also differed significantly by region, with the higher rates of ALND seen in the South (49.8% ALND in the South vs. 37.4%, 30.3%, and 42.3% in the West, Midwest, and Northeast, respectively, *p* < 0.001; Table S1).

### Factors Associated with Increased Odds of Axillary Lymph Node Dissection

In a multivariable logistic regression model, we accounted for race, age, region of treatment, tumor receptor subtype, cN status, ypN status, cT, and ypT (Table 5). We found that patients with cN+ disease had significantly higher odds of receiving ALND compared with those with cN- disease (OR 6.58, 95% confidence interval (CI) 4.86–8.98, *p* < 0.001). Similarly, ypN+ status was also associated with increased odds of ALND compared to ypN− status (OR 7.7, 95% CI 5.45–11.0, *p* < 0.001). Patients with ypT3-T4 tumors also had greater odds of undergoing ALND compared with those with ypTis/T0 disease (OR 2.42, 95% CI 1.37–4.32, *p *= 0.002). Treatment region was also a significant factor, with patients treated in the South having significantly higher odds of undergoing ALND compared with those treated in the West (OR 2.18, 95% CI 1.52–3.14, *p* < 0.001). When accounting for all of these factors, we found that Black patients remained more likely to undergo ALND compared to White patients (OR 1.7, 95% CI 1.09–2.66, *p *= 0.02).

**Table 5:** Multivariable logistic regression model for odds of sentinel lymph node surgery adjusting for race, age at screening, region, tumor subtype, clinical and pathological nodal status, clinical T, and pathological T stage

Variables	Odds ratio	95% Confidence interval	*p*
*Race*			
White	(reference)		
Black	1.7	1.09–2.66	0.02
Asian/other	1.26	0.77–2.07	0.4
Age	1.00	0.99–1.01	0.9
*Region*			
West	(reference)		
Midwest	0.77	0.53–1.12	0.2
Northeast	1.01	0.53–1.92	> 0.9
South	2.18	1.52–3.14	< 0.001
*Tumor subtype*			
HR+/HER2−	(reference)		
HR−/HER2−	1.18	0.84–1.67	0.3
HER2+	1.36	0.94–1.97	0.1
*cN status*			
Negative	(reference)		
Positive	6.58	4.86–8.98	< 0.001
*ypN status*			
Negative	(reference)		
Positive	7.7	5.45–11	< 0.001
*cT at diagnosis*			
T1-T2	(reference)		
T3-T4	1.3	5.45–11	< 0.001
*ypT*			
T0/Tis	(reference)		
T1-T2	1.03	0.73–1.45	0.9
T3-T4	2.42	1.37–4.32	0.002

*HR* hormone receptor; *HER2* human epidermal growth factor receptor 2; +, receptor status positive; −, receptor status negative; *c* clinical; *yp* pathologic; *Tis* in situ

## Discussion

In this analysis of the multicenter NAC trial I-SPY 2, we identified persistent race-based disparities in the surgical management of the axilla. Black-identifying patients had significantly higher rates of ALND compared with White and Asian/other patients. This disparity remained amongst those who achieved nodal downstaging from cN+ to ypN− following NAC, a subgroup in whom de-escalation of axillary surgery would typically be considered appropriate.^3,16,17^

Clinical trials are designed to standardize treatment and reduce variability in the delivery of care. Some investigators have found that Black women achieve lower rates of pCR compared with their counterparts.^18–20^ Others find no differences in pCR by race.^21,22^ A previous investigation of the I-SPY 2 trial found that there were no significant differences in rates of pCR by race.^14^ Also well-established in the literature, Black women have been shown to undergo increased rates of ALND compared to their counterparts.^11,12^ Relation et al. found that this disparity persists in the National Cancer Database, even in the context of NAC, where nodal downstaging would allow for more conservative axillary management.^15^ Our findings build upon this and show that even in the context of a controlled environment such as a clinical trial where no differences in pCR have been previously established, there exist differences in axillary management by race. This suggests that the lack of mandated surgery intervention in clinical trials can allow factors beyond clinicopathological characteristics to influence surgical decision-making.

Several factors could contribute to the observed disparity. Although our multivariable analysis adjusted for region, tumor subtype, clinical and pathologic nodal status, and tumor size, unmeasured institutional or provider-level differences may still play a role. Variations in regional practices may also contribute to the observed disparities, given higher rates of ALND in the South. However, even when adjusting for this, Black patients were more likely to undergo ALND, suggesting that regional variation in practice patterns alone do not account for the differential use of ALND by race. This suggests geographic differences in adherence to de-escalation practices may influence surgical decision-making independent of patient biology. Implicit bias in clinical decision-making is another possible explanation, potentially influencing whether providers feel comfortable omitting ALND. Black women have previously been shown to have more triple-negative tumors, advanced nodal involvement, higher rates of recurrence, and experience higher mortality, which could influence patient and provider treatment choices.^23–28^ Additionally, factors such as patient preferences, socioeconomic status, and comorbidities may all influence surgical decision-making. Reeder-Hayes et al. found that in early-stage breast cancer, educational attainment was not associated with type of axillary surgery, but patients enrolled in Medicaid had significantly lower odds of receiving SLN surgery (OR 0.61, 95% CI 0.47–0.78).^29^ Nayyar et al. found higher ALND use amongst those who were uninsured, and those who resided in areas with low income and low education levels compared to those with private health insurance and those with higher average income and education levels.^12^ Differences in insurance access, education status, and health literacy can contribute to less engagement during decision-making, leading to misunderstandings about the choice between ALND and SLN.^30^

The results of this study should be viewed in the context of its limitations. While we adjusted for numerous clinicopathological variables, our study did not capture factors that influence patient and surgeon choices. Although I-SPY 2 is a geographically diverse, multicenter trial, some of our racial subgroups were relatively small, which limited the statistical power to detect differences in these populations (Asian/other). Additionally, data on radiotherapy usage and BMI were unavailable. Access to postoperative radiotherapy could potentially influence ALND use, particularly for patients facing barriers to care. Body habitus may influence sentinel lymph node mapping rates, with prior studies suggesting mixed success rates in those with higher BMI.^31–33^ Post-NAC axillary staging information such as repeat ultrasound if performed or physical examination findings were not available for this analysis, but could also have contributed to surgical decision making. Finally, surgical management of the axilla is a rapidly evolving landscape, and our observed differences could be confounded by potential shifts in practice patterns over time.^17^

## Conclusions

Our findings revealed persistent race-based disparities in axillary management in I-SPY 2, highlighting the need for clearer, standardized guidelines to guide axillary management decisions after NAC. Future studies should consider the collection of more granular demographic data to better understand and address disparities in breast surgical oncology. Initiatives aimed at improving provider education, reducing bias, and ensuring equitable treatment plans are essential to advancing de-escalation strategies in breast cancer care.

## Supplementary Information

Below is the link to the electronic supplementary material.

Supplementary file1 (DOCX 18 KB)

## Data Availability

The datasets generated during and/or analyzed during the current study are not publicly available but are available from the corresponding author upon reasonable request.
