# Supplementary material for: Disparities in the Surgical Management of the Axilla by Self-Identified Race in the Multicenter Neoadjuvant I-SPY2 Trial
Source: Ann Surg Oncol. 2025 Jul 23;32(11):8211–9. doi: 10.1245/s10434-025-17864-y (PMC12494629; doi:10.1245/s10434-025-17864-y)
Supplement: Supplementary file 1 — Supplementary file1 (DOCX 18 KB) [file 10434_2025_17864_MOESM1_ESM.docx]

**Supplementary Tables**

**Table S1.** Axillary Surgery by Region

| Region | SLN-only (n=849) | ALND (n=545) | p-value |
| --- | --- | --- | --- |
| West^1^ | 421/673 (62.6%) | 252/673 (37.4%) | <0.001 |
| South^2^ | 167/333 (50.2%) | 166/333 (49.8%) |  |
| Midwest^3^ | 216/310 (69.7%) | 94/310 (30.3%) |  |
| Northeast^4^ | 45/78 (57.7%) | 33/78 (42.3%) |  |

SLN, sentinel lymph node surgery; ALND, axillary lymph node dissection. N=1,394.

**Table S2.** Axillary Surgery by Region, Race, and Nodal Status

| Nodal Status | Race | Region | SLN-only (n=769) | ALND (n=494) |
| --- | --- | --- | --- | --- |
| cN- | Black |  |  |  |
|  |  | West | 9/13 (69.2%) | 4/13 (30.8%) |
|  |  | South | 31/35 (88.6%) | 4/35 (11.4%) |
|  |  | Midwest | 9/12 (75.0%) | 3/12 (25.0%) |
|  |  | Northeast | 5/5 (100.0%) | 0/5 (0.0%) |
|  | White |  |  |  |
|  |  | West | 214/258 (82.9%) | 44/258 (17.1%) |
|  |  | South | 94/112 (83.9%) | 18/112 (16.1%) |
|  |  | Midwest | 125/134 (93.3%) | 9/134 (6.7%) |
|  |  | Northeast | 22/23 (95.7%) | 1/23 (4.3%) |
| cN+ | Black |  |  |  |
|  |  | West | 6/12 (50.0%) | 6/12 (50.0%) |
|  |  | South | 8/37 (21.6%) | 29/37 (78.4%) |
|  |  | Midwest | 9/20 (45.0%) | 11/20 (55.0%) |
|  |  | Northeast | 0/22 (0.0%) | 22/22 (100.0%) |
|  | White |  |  |  |
|  |  | West | 128/287 (44.6%) | 159/287 (55.4%) |
|  |  | South | 28/138 (20.3%) | 110/138 (79.7%) |
|  |  | Midwest | 63/129 (48.8%) | 66/129 (51.2%) |
|  |  | Northeast | 18/26 (69.2%) | 8/26 (30.8%) |
| ypN- | Black |  |  |  |
|  |  | West | 14/16 (87.5%) | 2/16 (12.5%) |
|  |  | South | 35/53 (66.0%) | 18/53 (34.0%) |
|  |  | Midwest | 16/20 (80.0%) | 4/20 (20.0%) |
|  |  | Northeast | 5/16 (31.3%) | 11/16 (68.8%) |
|  | White |  |  |  |
|  |  | West | 284/349 (81.4%) | 65/349 (18.6%) |
|  |  | South | 115/162 (71.0%) | 47/162 (29.0%) |
|  |  | Midwest | 171/196 (87.3%) | 25/196 (12.7%) |
|  |  | Northeast | 30/36 (83.3%) | 6/36 (16.7%) |
| ypN+ | Black |  |  |  |
|  |  | West | 1/9 (11.1%) | 8/9 (88.9%) |
|  |  | South | 4/19 (21.1%) | 15/19 (78.9%) |
|  |  | Midwest | 2/12 (16.7%) | 10/12 (83.3%) |
|  |  | Northeast | 0/11 (0.0%) | 11/11 (100.0%) |
|  | White |  |  |  |
|  |  | West | 58/196 (29.6%) | 138/196 (70.4%) |
|  |  | South | 7/88 (7.9%) | 81/88 (92.1%) |
|  |  | Midwest | 17/67 (25.4%) | 50/67 (74.6%) |
|  |  | Northeast | 10/13 (76.9%) | 3/13 (23.1%) |

SLN, sentinel lymph node surgery; ALND, axillary lymph node dissection; c, clinical; yp, pathologic. N=1,263.
